# Association between mannose-binding lectin variants, haplotypes and risk of hepatocellular carcinoma: A case-control study

**DOI:** 10.1038/srep32147

**Published:** 2016-08-25

**Authors:** Chenghao Su, Yong Lin, Lin Cai, Qianguo Mao, Jianjun Niu

**Affiliations:** 1 Xiamen Center for Disease Control and Prevention, Xiamen, Fujian Province, China; 2 School of Public Health, Xiamen University, Xiamen, Fujian Province, China; 3 School of Public Health, Fujian Medical University, Fuzhou, Fujian Province, China; 4 Department of hepatology, Xiamen Hospital of Traditional Chinese Medicine, Xiamen, Fujian Province, China; 5 Zhongshan Hospital, Xiamen University, Xiamen, Fujian Province, China

## Abstract

The innate immunity gene mannose-binding lectin2 (MBL2) has played an important role in hepatitis B virus (HBV) infection, and the relationship between MBL2 variants and hepatocellular carcinoma (HCC) risk has not yet been identified. In total, 315 HCC cases and 315 healthy controls were enrolled and blood samples were acquired. High resolution melt analysis (HRM) was employed to genotype 6 polymorphisms in MBL2 gene. Increased HCC risk in carriers of LL genotype of −550 polymorphism with an adjusted OR (AOR) of 1.61 (95%CI = 1.00–2.57) was observed but no significant association detected in HL genotype. Both YX and XX genotype demonstrated a significantly elevated HCC risk in the analysis of −221 polymorphism. The B variants in codon 54 was also significantly associated with elevated HCC risk. HYB was identified as the protective factor of HCC while LXB was significantly associated with increase HCC risk. ELISA technique revealed that the MBL2 protein was significantly reduced in HCC cases. Moreover, both IL-1β and IL-6 were inversely associated with plasma MBL2 level.The mutations in MBL2 could lead to compromised innate immunity, and possibly lead to elevated HCC risk, and a novel haplotype HXB has been identified with a rate of 12.5%.

In worldwide range, the incidence of hepatocellular carcinoma (HCC) ranked fifth and also maintained the second position in mortality, it is estimated that there would be 7,825,000 newly diagnosed HCC cases and 7,455,000 HCC caused death in 2012, and approximately 50% of incidence and mortality would occurred in China^1^. Due to the poor prognosis and absence of effective treatment, the mortality of HCC is relatively high when comparing with other cancers, and consequently has become a serious public health issue in China. The chronic infection of hepatitis B virus and hepatitis C virus has been identified as the major risk factor of developing HCC, however, the development of HCC is a process involves multi-factors and complicated process, therefore, aside of viral factors and aflatoxin^2^, genetic factors can also alter the risk of HCC in population. Although the activation of oncogene and deactivation of tumor suppressors have been recognized as an important mechanism in developing cancer, and the results from both laboratory and epidemiology studies have confirmed the association. For example, the mutation in codon 72 of P53 would compromise the activity of its protein, and consequently, leads to the elevation of cancer risk. A case-control study in Morocco observed an elevated HCC risk among population with homozygote of codon 72 mutation, especially in women^3^.

The polymorphism in immune related genes can also contribute to the development of cancer, for instance, in global range there were only 3% to 8% of patients with hepatitis B and 1% to7% of patients with hepatitis C eventually progressed to HCC^4^, which indicated that the variants in immune related genes, and virus would lead to the susceptibility difference among population. Mannose binding lectin 2 gene is located in the human chromosome 10, the production plays a critical role in first-line defense against pathogens by activating complement system through lectin pathway. Previous study revealed that there were 6 polymorphisms in MBL2 gene closely associated with the serum level of MBL2^5^, among these variants, 2 of them are located in promoter, 1 in untranslated region, and the 3 remaining in exon 1. The above mentioned variants and its haplotypes are capable of effecting the serum MBL2 level significantly, and maintaining appropriate level of MBL2 is of critical importance in combating infection for human body. So far seldom studies on the association between MBL2 polymorphisms and HCC risk were reported, only a study conducted in Italian population found no significant association between MBL2 genotypes and HCC risk^6^, however, the study has limited sample size, and it performed stratification according to both hepatitis B and C infection, which led to reduction in both sample size and statistical power. Therefore, in order to reveal the association in MBL2 polymorphisms and HCC risk, we have conducted a case-control study in southeast China.

## Results

### Demographic and clinical characteristics

As can be seen in Table 1, no significance observed among HCC cases and healthy controls in terms of age (*P* = 1.000), gender (*P* = 1.000) and ethnicity (*P* = 0.499). However, we observed significant difference in education and HBsAg between cases and controls with a *P* value smaller than 0.001. In addition, the clinical features of all HCC cases, including tumor size, cirrhosis, vascular invasion, and metastasis in the day of admission were also reported in the same table.

### Conditional logistic regression of MBL2 polymorphisms

The HRM analysis did not detect any mutation in codon 52 and 57 among all subjects, which is consistent with the genetic profile of Asian population. Therefore, we calculated the OR and 95%CI in the rest of 4 polymorphisms by using conditional logistic regression, and the results were presented in Table 2. After adjusted for education, we observed an increased HCC risk in carriers of LL genotype of −550 polymorphism with an AOR of 1.61 (95%CI = 1.00–2.57), but no significant association detected in HL genotype. Both YX and XX genotype demonstrated a significantly elevated HCC risk, and the AORs were 1.51 (95%CI = 1.03–2.21) and 5.67 (95%CI = 1.82–17.67), respectively. The analysis did not found any significance association in +4 polymorphism and HCC risk, both in PQ and QQ genotype. As for codon 54, both AB and BB genotype were significantly correlated with elevated HCC risk, with AOR of 1.48 (95%CI = 1.01–2.17) and 3.99 (95%CI = 1.55–10.30), respectively.

### MBL2 haplotypes and the HCC risk

R package haplo.stats were used to estimate the frequency of MBL2 haplotypes among subjects, the results showed that LYB was the most frequent one in cases and controls with frequency of 40.6% and 38.5%, respectively. In the second and the third position were HYB and HXB. We considered that haplotype with frequency lower than 2% in overall subjects as rare haplotype and would be excluded in generalized linear model. The most frequent haplotype LYB was employed as reference category, and the OR and 95%CI were estimated by comparing with haplotype LYB. Among all included haplotypes, HYB was identified as the protective factor of HCC with an OR of 0.64 (95%CI = 0.49–0.85), and LXB was significantly associated with increase HCC risk, the OR for LXB carriers was 2.75 (95%CI = 1.13–6.64), the detailed results were presented in Table 3.

### Plasma MBL2 level and the MBL2 polymorphisms

**Figure 1:**
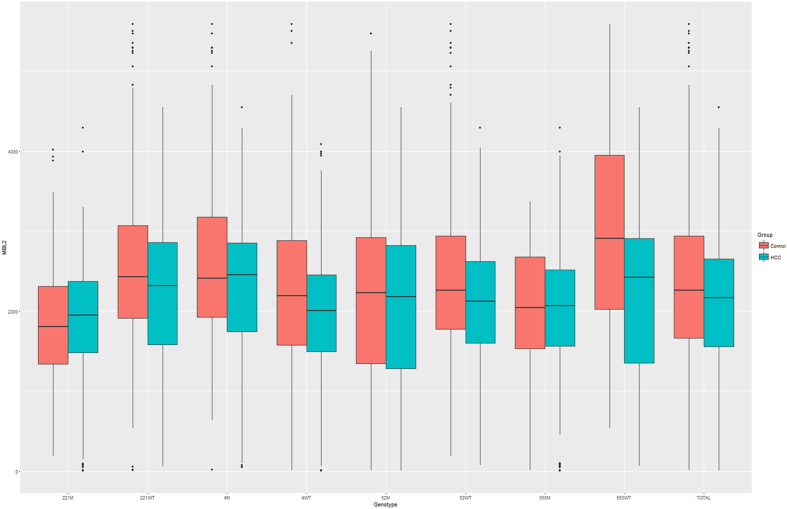
The comparison on MBL2 polymorphisms and plasma MBL2 concentration in HCC cases and healthy controls.

As presented in Table 4 and Fig. 1, the median of plasma MBL2 level in HCC cases was 2167 ng/ml, while the corresponding level in controls was 2257 ng/ml.The results of Mann-Whitney’s U test suggested that the difference was significant, indicating that the MBL2 level was significantly suppressed in HCC cases when comparing with the healthy controls (*P* = 0.004). In the stratification analysis by genotype, we found that the MBL2 level was significantly different between two groups among subjects with wild type of 4 MBL2 polymorphisms we investigated. Similar with the previous results, the MBL2 level was significantly higher in healthy controls than HCC cases, however, such difference was not observed among the subjects carrying mutated MBL2 polymorphisms.

### The correlation between MBL2 level and other 2 cytokines

In Table 5, the median of other 2 cytokines in HCC cases and controls was reported, and statistical comparison was made by using Mann-Whitney’s U test. The results showed that both IL-1β and IL-6 were significantly elevated in HCC cases when comparing with healthy controls, and the *P* values were <0.001 and 0.002, respectively. Furthermore, the Spearman’s rank correlation found that the MBL2 level was inversely associated with the level of IL-1β and IL-6. Among two cytokines, the association between MBL2 and IL-1β was stronger, as it has higher correlation coefficient which reached −0.977. As for the IL-6, the coefficient between it and MBL2 level was −0.770, and the *P* value was less than 0.001, suggesting that the association was of significance.

## Discussion

The studies regarding the association between MBL2 polymorphisms and cancer risk have been seldom reported, as for hepatocellular carcinoma, only a study conducted in Italian population investigated the relationship between polymorphisms in the exon 1 of MBL2 and HCC risk. This study performed analysis in subjects divide into 4 groups in accordance with infection status of hepatitis B and C, and found no significant association in all groups^6^. We considered that the insignificant result was due to limited sample size and the study design further diminished the statistical power. Moreover, this study only investigates the polymorphisms in exon 1, and the polymorphisms in promoter region and untranslated region (UTR) have not been included, therefore, it was impossible to study the MBL2 haplotype based on the data acquired. The main strength of our study was that we performed HRM analysis to determine the genotype of all 6 MBL2 polymorphisms among 630 subjects in total, and we found no mutation on the codon 52 and 57, which is consistent with the genetic profile of Asian population^7^, and these results also demonstrated the HRM analysis we used in this study was solid.

The conditional logistic analysis revealed that both 2 polymorphisms in promoter region can alter the HCC risk significantly. Although it is generally acknowledged that the variants in exon 1 have greater impact in serum MBL2 level, mutations on promoter region can change the serum level as well. As the matter of fact, the MBL2 gene possess 2 promoters which are located in the upstream of exon 1, normally the transcription of MBL2 gene is initiated in promoter 1, however, approximately 10–15% of transcription is activated by promoter 0, which will produce MBL protein with an additional 5′ untranslated part encoded by an extra exon 0^8^. The promoter 1 regulates most part of MBL2 transcription, therefore, the polymorphisms in promoter 1 would significantly affect the serum level of MBL2, and among 2 variants identified, it is suggested that −221 Y/X play a more important role. For example, −221 Y/X has been linked with chronic infection in some clinical studies, in addition, a genome wide association study reported the association between −221 Y/X polymorphism and the susceptibility of leprosy among Chinese population^9^. Our analysis observed the positive correlation between both −550 and −221 polymorphisms and the risk of HCC, the adjusted ORs were 1.61 (95%CI = 1.00–2.57) and 5.67 (95%CI = 1.82–17.67), respectively. As we can see the OR for −221 Y/X was greater than the corresponding indicator of −550 H/L, which is consistent with previous studies.

The polymorphisms in exon 1 are capable of affecting the serum MBL2 level more than the variants in promoter, because all 3 variants would all lead to missense mutation, and consequently the amino acid substitution would cause the instability of peptide, and the production would soon be degraded. It is reported that the exon 1 variants are very common in population, especially in Caucasians, in detail, about 40% of Caucasians carries at least one mutation in exon 1^10^. Our HRM analysis observed no D and C variants in all subjects, and the frequency of B variant heterozygote and homozygote were 24.9% and 4.3%, respectively, which is also consistent with genetic profile of Asian population.It is reported that B homozygote carriers have only 1/10 serum MBL2 level when comparing with wild type, and our analysis showed that the adjust ORs for B variant heterozygote and homozygote were 1.48 (95%CI = 1.01–2.17) and 3.99 (95%CI = 1.55–10.30), respectively.

As for the haplotypes, the previous study revealed that the haplotype HY, LY and LX were associated with high, mediate and low serum MBL2 level correspondingly^10^, and the mutation frequency of MBL2 polymorphisms vary with ethnicity. What need to notice is that we identified a novel haplotype HX which was seldom reported previously. The haplotype analysis by R package haplo.stats estimated that the frequency of HXB among all subjects reached 12.5%, which was deviated data acquired from other populations. In generalized linear model, the hapotype LXB was associated with increased HCC risk with an OR of 2.75 (95%CI = 1.13–6.64). As for the novel haplotype HXB, the OR was 1.22 (95%CI = 0.84–1.78), the difference was not significant, although it reached the margin of significance. As previously stated, the frequency of haplotype HXB reached 12.5% in total study population, therefore, it is of critical importance to study the function of HXB.

In our study, we managed to perform the determination of plasma MBL2 level in all subjects,as it is generally acknowledged that the polymorphisms in MBL2 gene is capable of altering the concentration which is the scientific basis of this study, therefore, statistical comparison has been made between two groups with same genotype. For the comparison on MBL2 level between different genotypes, we presented it as Supplementary materials, and the results were consistent with previous study^11^. The overall comparison on MBL2 level between HCC cases and controls revealed that the MBL2 level was reduced in cases group, and the result was as we expected. But strikingly, the reduction on MBL2 level in HCC cases can only be observed among subjects with wild type of polymorphisms, no significant difference was observed in subjects with mutation which was consistent in all 4 polymorphisms we investigated. The reduction on MBL2 level in cancer patients has been confirmed among 261 colon cancer cases and 537 controls in United States^12^, and the ethnicity also has impact on the MBL2 level.

Our study also revealed that −550 and −221 polymorphisms in promoter region and codon 54 B mutation were correlated with elevated HCC risk among study subjects, it is generally acknowledged that all these polymorphisms are contributed to the reduction on serum MBL2 level, therefore, it can be assume that the reduced MBL2 level could possibly be a risk factor of HCC. Combing these consistent results both in genotype and concentration analysis together, we can assume that higher MBL2 level and may has protective effect against the onset of HCC by enhancing immunological surveillance against hepatocellular carcinogenesis. The one underlying mechanism of the protective effect may work by recognizing and binding to oligosaccharide ligands expressed on the surfaces on human tumor cells^13^. But interestingly, although mutant MBL2 protein loses its capability of activating complement system, however, it still possesses antitumor activity like normal MBL2 protein does. Animal experiment compared the cytotoxicity between mice with normal MBL2 gene and mutant MBL2, and the results found no significant difference, suggesting the anti-tumor activity of MBL2 is cell-mediated^14^. Therefore, we can consider that the altered HCC risk caused by MBL2 polymorphisms were not triggered by its anti-tumor activity but due to the mutant MBL2 protein failed to activate complement system, and eventually lead to the deficiency of innate immunity. As can be seen, the HBsAg positive rate for HCC cases and healthy controls were 70.2% and 12.7%, respectively. The hepatitis B virus is a kind of mannooligosaccharide-bearing pathogens, therefore, MBL2 protein can recognizes and binds specifically to HBV and initialize the procedure of clearing virus, however, mutant MBL2 protein possesses no such activity, and we can assume that compromised innate immunity would lead to more severe progression of chronic HBV infection.

Additionally, both IL-6 and IL-1β were significantly elevated in HCC cases when comparing with control subjects. This result was reasonable, because both two cytokines possess the property of pro-inflammatory^15^,^16^, and inflammation is commonly occurred during the development and progression of cancer.The difference on plasma cytokines level between two groups can also attributed to the pain that cancer causes, an animal experiment showed that both IL-6 and IL-1β were elevated on the 3^rd^ day after operation, and can change by the intensity of pain^17^. Moreover, we observed the inverse association between MBL2 and cytokines level, a previous report on the MBL2 level and Neisseria meningitidis also supported our findings, in an *ex vivo* model, the high MBL2 level profoundly suppressed the production of inflammatory cytokines, including IL-6, IL-1β and TNF-α, while MBL2 deficiency which is less effective in activating complement system could lead to the over expression of IL-6 and IL-1β^18^. Although the underlying mechanism was not clear, but we assume that maintaining high MBL2 level may reduce the inflammatory damage and can inhibit the progression of cancer. Besides, our study also identified a novel haplotype HX, and the function and its influence on serum MBL2 level have not yet been investigated, so further functional study on haplotype HX is still needed.

## Materials and Methods

### Study subjects

Consecutive primary HCC cases (ICD9-155) were recruited from Xiamen Zhongshan Hospital and Xiamen Traditional Chinese Medicine Hospital between Dec 2011to Jul 2014. All HCC patients were incidence patients with the confirmation of liver biopsy and were permanent residents who lived in Xiamen over 10 years and aging from 20 to 79 years old. Patients were excluded if any of the following conditions were met: (1) liver disease due to parasitosis, diabetes, fatty liver, metabolism disorders or severe cardiovascular diseases; (2) presence of tumors other than HCC; (3) autoimmune hepatitis or toxic hepatitis; (4) refusal or inability to participate in the investigation because of critical status.

Community-based healthy controls was pair matched with HCC cases by age (±3 years) and gender, recruited from the people who attending physical examination in the community healthcare center. All control subjects were also permanent residents of Xiamen city without prior history of any cancer aging from 20 to 79 years old. In order to diminish possible risk of bias at maximum level, we have randomly selected these control subjects and confirmed that all study subjects were ethnically unrelated.

In total, 315 incidence HCC cases and identical number of healthy controls were enrolled in our study. All study subjects have physically signed a written informed consent, and all experiments involved in our study are in agreement with the Helsinki declaration and the Ethic Committee of Xiamen Center for Disease Control and Prevention has approved all procedures of this study.

### Clinical characteristics

The clinical characteristics of each subject with HCC, including tumor size, liver cirrhosis, invasion, and metastasiswere obtained by reviewing medical record of hospital. Two investigators performed the data entry independently by using Epidata software 3.0 and double entry verification was conducted to lower the chance of error.

### Determination of hepatitis B infection

All cases and healthy controls provided a 5-ml blood sample on the day of interview. Blood samples were centrifuged at 4000 rpm for 10 min to separate plasma and blood cells and stored at −78 °C prior to determination of serological marker for hepatitis B infection. The laboratory assay was performed in the plasma sample of each study participant by using commercialenzyme-linked immunosorbent assay kits (WantaiBioPharm, Beijing, China). All procedures were strictly conformed with manufacture’s manual, in addition, approximately 5% of the samples were randomly selected and repeated for test as duplicated controls.

### Determination of MBL2 polymorphisms

Human genomic DNA was extracted from blood cells using the MagNA Pure LC DNA Isolation Kit I (Roche Applied Science, Mannheim, Germany). HRM analysis were performed to determine the 6 polymorphisms in MBL2 gene, namely −550 H/L, −221 Y/X, +4 P/Q, codon 52, 54 and 57. Due to the close physical distance between codon 52 and 54, we managed to use single PCR reaction to genotype them both, therefore, 5 primer sets were used to determine 6 above-mentioned MBL2 polymorphisms (See Table 6). Each PCR mixture containing PCR buffer 1×, 2.0 mM of Magnesium chloride, 0.2 mM of dNTP, 0.08 μM of forward primer, 0.8 μM of reverse primer, and 1 U of Taq polymerase to a total volume of 25 μL. The real-time PCR was conducted by using CFX-96 (BioRad, Hercules, CA, USA) with an initial denaturation step of 95 °C for 10 min, followed by 49 cycles of 95 °C for 15 sec, 55 °C for 15 sec, and 76 °C for 20 sec. Data acquisition was performed between 40 °C to 84.4 °C at the rate of 0.4 °C. Table 7 presents the melt peak of each genotype of MBL2 polymorphisms we studied in this study, 10% of sample were randomly tested for quality control.

### Determination of plasma level of MBL2, IL-6 and IL-1β

Human MBL2, IL-6 and IL-1β quantikine ELISA kits (R&D systems, Minneapolis, MN, USA) were used to determine the plasma level of corresponding protein. Following is the detailed procedure for MBL2 determination: (1) Firstly pipette 50 μl of assay diluent (50 μl for IL-1β; 100 μl for IL-6) to each well. (2) Then pipette 50 μl of standard, control or plasma sample (200 μl for IL-1β; 100 μl for IL-6) to each well, cover with plate sealer and incubate at room temperature for 2 hours. (3) Aspirate each well and wash, repeating the process 3 times for a total of 4 washes. (4) Add 100 μL of Conjugate (200 μl for IL-1β and IL-6) to each well. Cover with a new plate sealer, and incubate at room temperature for 2 hours on the shaker. (5) Aspirate and wash for 4 times. (6) Add 100 μL Substrate Solution (200 μL for IL-1β and IL-6) to each well. Incubate at room temperature for 30 minutes on the benchtop, and keep from the exposure of light. (7) Add 100 μL of Stop Solution (50 μL for IL-1β and IL-6) to each well. Read at 450 nm within 30 minutes. Set wavelength correction to 540 nm or 570 nm. (8) Using the software provided by manufacturer to draw a standard curve and read the protein concentrations for each sample.

### Statistical Analysis

Chi-squared test was conducted to examine the differences in demographic characteristics and potential confounders between HCC cases and healthy controls. Conditional logistic regression was applied to determine the association between MBL2 polymorphisms and the risk of developing HCC, odd ratios (ORs) and 95% confidence intervals (95%CI) were calculated. R Package Haplo.stats was used to estimate MBL2 haplotypes and generalized linear model was employed to calculate the HCC risk of each haplotype.The comparison of non-normally distributed data, including plasma level of MBL2, IL-1β and IL-6 was conducted by using Mann-Whitney’s U test. The Spearman’s rank correlation was used to estimate the coefficient between MBL2 level and other two cytokines.A 2-tailed *P* value of <0.05 was accepted as being statistically significant. Expect haplotype analysis, all other statistical analyses were performed by using SPSS software (IBM, Chicago, IL, USA) version 19.

## Additional Information

**How to cite this article**: Su, C. *et al*. Association between mannose-binding lectin variants, haplotypes and risk of hepatocellular carcinoma: A case-control study. *Sci. Rep.* **6**, 32147; doi: 10.1038/srep32147 (2016).

## Supplementary Material

Supplementary Information

## Figures and Tables

**Table 1:** The demographic and clinical characteristics of HCC cases and healthy controls.

Variables	HCC Cases	Healthy Controls	*χ*^*2*^	*P*
Age				
≤30 Years	9 (2.9)	9 (2.9)		
31–50 Years	94 (29.8)	94 (29.8)		
51–70 Years	166 (52.7)	166 (52.7)		
≥71 Years	46 (14.6)	46 (14.6)	0.00	1.000
Gender				
Male	264 (83.8)	264 (83.8)		
Female	54 (16.2)	54 (16.2)	0.00	1.000
Ethnicity				
Han	315 (100.0)	313 (99.4)		
Others	0 (0.0)	2 (0.6)	—	0.499^#^
Education				
Elementary school	173 (54.9)	149 (47.3)		
Middle school	127 (40.3)	123 (39.0)		
College or above	15 (4.8)	43 (13.7)	15.37	<0.001^*^
HBsAg				
Negative	94 (29.8)	275 (87.3)		
Positive	221 (70.2)	40 (12.7)	214.30	<0.001^*^
Tumor size				
≤ 5 cm	150 (47.6)	—		
> 5 cm	165 (52.4)	—	—	—
Cirrhosis				
No	157 (49.8)	—		
Yes	158 (50.2)	—	—	
Vascular invasion				
No	223 (70.7)	—		
Yes	92 (29.3)	—	—	
Metastasis				
No	210 (66.7)	—		
Yes	105 (33.3)	—	—	

*P < 0.05.

**Table 2:** The association between MBL2 polymorphisms and HCC risk.

MBL2 polymorphism	HCC cases	Healthy controls	AOR (95%*CI*)^#^
−550 H/L			
HH	82 (26.0)	115 (36.5)	1.00 (Ref)
HL	159 (50.5)	141 (44.8)	1.48 (1.00–2.19)
LL	74 (23.5)	59 (18.7)	1.61 (1.00–2.57)^*^
−221 Y/X			
YY	207 (65.7)	239 (75.9)	1.00 (Ref)
YX	91 (28.9)	72 (22.9)	1.51 (1.03–2.21)^*^
XX	17 (5.4)	4 (1.3)	5.67 (1.82–17.67)^*^
+4 P/Q			
PP	214 (67.9)	228 (72.4)	1.00 (Ref)
PQ	88 (27.9)	81 (25.7)	1.18 (0.82–1.70)
QQ	13 (4.1)	6 (1.9)	2.51 (0.94–6.74)
codon 54			
AA	207 (65.7)	239 (75.9)	1.00 (Ref)
AB	88 (27.9)	69 (21.9)	1.48 (1.01–2.17)^*^
BB	20 (6.3)	7 (2.2)	3.99 (1.55–10.30)^*^

*P < 0.05.

^#^Adjusted by education.

^#^Calculated by Fisher’s exact.

**Table 3:** The association between MBL2 haplotypes and HCC risk.

Haplotype	HCC cases	Healthy controls	OR (95%*CI*)	coefficient	*P*
LYB	40.6%	38.5%	1.00 (Ref)	—	—
HYA	11.6%	9.5%	1.18 (0.76–1.82)	−0.001	0.168
HYB	25.6%	38.2%	0.64 (0.49–0.85)^*^	−0.48	0.002
LXA	0.0%	0.2%	—	—	—
LXB	5.7%	1.3%	2.75 (1.13–6.64)^*^	1.33	0.00001
LYA	2.4%	1.2%	—	—	—
HXB	14.1%	11.1%	1.22 (0.84–1.78)	0.27	0.110

*P < 0.05.

**Table 4:** The association between MBL2 polymorphisms and plasma MBL2 concentration in HCC cases and healthy controls.

MBL2 polymorphism	Median(ng/ml)		Mean Rank		*P*
	HCC cases	Healthy controls	HCC cases	Healthy controls	
−550 HH	2421.00	2906.00	81.26	111.65	<0.001^*^
HL/LL	2065.00	2402.00	218.14	216.03	0.861
−221 YY	2314.00	2426.00	205.41	239.17	0.006^*^
YX/XX	1947.50	1807.00	93.75	90.72	0.703
+4 PP	2004.50	2188.50	203.09	238.78	0.003^*^
PQ/QQ	2455.00	2412.00	99.79	89.95	0.216
codon 54 A	2124.00	2257.00	204.89	239.62	0.005^*^
AB/BB	2226.00	2179.00	89.41	96.89	0.348
Total	2167.00	2257.00	294.46	336.54	0.004^*^

*P < 0.05.

**Table 5:** The plasma level of IL-1β, IL-6 and the correlation with MBL2.

Cytokines	Median (pg/ml)		*P*^*#*^	Spearman Correlation with MBL2	*P*
	HCC cases	Healthy controls			
IL-1β	4.55	5.36	<0.001	−0.977	<0.001^*^
IL-6	8.08	1.89	0.002	−0.770	<0.001^*^

^#^*P* value for Mann-Whitney’s U test.

*P < 0.05.

**Table 6:** The primer and probe sequence for high-resolution melt analysis.

MBL2 ploymorphsim	Forward Primer (5′→3′)	Reverse Primer (5′→3′)	Probe (5′→3′)^*^
−550 H/L	TCTCAACCTCAGATCAACCTC	CCAACGTAGTAAGAAATTTCCAGAG	ROX-TTGGTGTTTTACACAGGCTTGCCTG-BHQ2
−221 Y/X	AGACACCTGGCGTTGCT	CCCTAAGCTAACAGGCATAAG	ROX-TAAACATGCTTTCCGTGGCAGTGAG-BHQ2
+4 P/Q	CCTCACCTTGGTGTGA GAA	CATCTATTTCTATATAGCCTGCACC	ROX-CACATATTTACCGAGCATGCCCTCTG-BHQ2
codon 52	TAGCTCTCCAGGCATCAAC	CAGAGACAGAACAGCCCA	ROX-AAGATGGGCGTGATGGCACC-BHQ2
codon 54			
codon 57	TAGCTCTCCAGGCATCAAC	CAGAGACAGAACAGCCCA	ROX-AGGGAGAAAAGGGGGAACCAGG-BHQ2

**Table 7:** Melting profiles for MBL2 polymorphisms.

MBL2 polymorphism	Genotype	Melt peak
−550 H/L	HH	56.8 °C ± 1 °C
	HL	66.2 °C ± 1 °C and 56.8 °C ± 1 °C
	LL	66.2 °C ± 1 °C
−221 Y/X	YY	58.9 °C ± 1 °C
	YX	67.5 °C ± 1 °C and 58.9 °C ± 1 °C
	XX	67.5 °C ± 1 °C
+4 P/Q	PP	68.3 °C ± 1 °C
	PQ	68.3 °C ± 1 °C and 64.2 °C ± 1 °C
	QQ	64.2 °C ± 1 °C
codon 52	AA	65.8 °C ± 1 °C
	AD	65.8 °C ± 1 °C and 56.5 °C ± 1 °C
	DD	56.5 °C ± 1 °C
codon 54	AA	65.8 °C ± 1 °C
	AB	65.8 °C ± 1 °C and 60.3 °C ± 1 °C
	BB	60.3 °C ± 1 °C
Codon 57	AA	64.8 °C ± 1 °C
	AC	64.8 °C ± 1 °C and 61.5 °C ± 1 °C
	CC	61.5 °C ± 1 °C
