# Supplementary material for: Association between mannose-binding lectin variants, haplotypes and risk of hepatocellular carcinoma: A case-control study
Source: Sci Rep. 2016 Aug 25;6:32147. doi: 10.1038/srep32147 (PMC4997250; doi:10.1038/srep32147)
Supplement: Supplementary Information [file srep32147-s1.doc]

Association between mannose-binding lectin variants, haplotypes and risk of hepatocellular carcinoma: A case-control study

*Chenghao Su,^1,2#^, Yong Lin,^1,3#^, Lin Cai^3^, Qianguo Mao^4^, Jianjun Niu^5*^*

1Xiamen Center for Disease Control and Prevention, Xiamen, Fujian Province, China

2School of Public Health, Xiamen University, Xiamen, Fujian Province, China

3School of Public Health, Fujian Medical University, Fuzhou, Fujian Province, China

4Department of hepatology, Xiamen Hospital of Traditional Chinese Medicine, Xiamen, Fujian Province, China

5Zhongshan Hospital, Xiamen University, Xiamen, Fujian Province, China

^#^These authors have equally contributed to this work

*Corresponding Author. E-mail:[niujianjun62@163.com](mailto:niujianjun62@163.com); Address: 209 Hubin South Road, Xiamen, Fujian Province, 361004,China.

**Supplemental materials**

Table S1 The association between MBL2 polymorphisms and plasma MBL2 concentration

| MBL2 polymorphism | Median(ng/ml) | Mean Rank | *P* |
| --- | --- | --- | --- |
| -550 HH | 2629.00 | 388.01 | - |
| HL/LL | 2062.00 | 282.51 | <0.001^*^ |
| -221 YY | 2351.50 | 345.44 | - |
| YX/XX | 1875.50 | 242.93 | <0.001^*^ |
| +4 PP | 2191.00 | 300.63 | - |
| PQ/QQ | 2204.00 | 321.63 | 0.188 |
| codon 54 A | 2416.00 | 358.39 | - |
| AB/BB | 2115.00 | 297.26 | <0.001^*^ |

**P<0.05*
